# Supplementary material for: Racial, ethnic, and age disparities in the association of mental health symptoms and polysubstance use among persons in HIV care
Source: PLoS One. 2023 Nov 28;18(11):e0294483. doi: 10.1371/journal.pone.0294483 (PMC10684077; doi:10.1371/journal.pone.0294483)
Supplement: S1 Table — (DOCX) [file pone.0294483.s001.docx]

# S1 Table. Characteristics of 4134 persons with HIV eligible for screening, stratified by screening completion, Kaiser Permanente Northern California, 2018–2020.

| Characteristic | Completed ≥1 screen  N = 2865 | No screen completed  N = 1269 |
| --- | --- | --- |
| Men | 2629 (92%) | 1152 (91%) |
| Race and ethnicity |  |  |
| Asian or Pacific Islander | 198 (7%) | 88 (7%) |
| Black | 558 (19%) | 278 (22%) |
| Hispanic | 419 (15%) | 277 (22%) |
| White | 1602 (56%) | 574 (45%) |
| Other/unknown | 88 (3%) | 52 (4%) |
| Age, years | 54 (13) | 51 (13) |
| HIV risk group |  |  |
| MSM | 2170 (76%) | 922 (73%) |
| IDU | 185 (6%) | 103 (8%) |
| Heterosexual or other | 510 (18%) | 244 (19%) |
| CD4 count, ^a^ cells/µL | 674 (306) | 639 (302) |
| HIV RNA <200 copies/mL ^a^ | 2678 (96%) | 1111 (93%) |
| Insurance type |  |  |
| Private | 1968 (69%) | 885 (70%) |
| Medicare | 732 (26%) | 287 (23%) |
| Medicaid | 142 (5%) | 85 (7%) |
| Other | 23 (1%) | 12 (1%) |
| NDI quartile ^b^ |  |  |
| 1 (least deprived) | 717 (25%) | 315 (25%) |
| 2 | 726 (25%) | 319 (25%) |
| 3 | 704 (25%) | 317 (25%) |
| 4 (most deprived) | 712 (25%) | 317 (25%) |

Numbers are N (%) or mean (SD). Abbreviations: IDU, injection drug use; MSM, men who have sex with men; NDI, neighborhood deprivation index; SD, standard deviation.

^a^ Closest measurement within six months before or after screening date, or first eligible visit for patients with no completed screen.

^b^ Calculated according to Messer et al. (2006) and divided in quartiles based on the distribution of the entire patient sample.
