# Supplementary material for: Racial, ethnic, and age disparities in the association of mental health symptoms and polysubstance use among persons in HIV care
Source: PLoS One. 2023 Nov 28;18(11):e0294483. doi: 10.1371/journal.pone.0294483 (PMC10684077; doi:10.1371/journal.pone.0294483)
Supplement: S2 Table — (DOCX) [file pone.0294483.s002.docx]

# S2 Table. Demographic and clinical characteristics stratified by race and ethnicity.

| Characteristic | Asian or Pacific Islander  N = 198 | Black  N = 558 | Hispanic  N = 419 | White  N = 1602 | Other/unknown  N = 88 |
| --- | --- | --- | --- | --- | --- |
| Men | 177 (89%) | 442 (79%) | 391 (93%) | 1538 (96%) | 81 (92%) |
| Age, years | 48 (39, 54) | 53 (41, 60) | 49 (40, 57) | 58 (51, 65) | 46 (35, 56) |
| HIV risk group |  |  |  |  |  |
| MSM | 151 (76%) | 325 (58%) | 338 (81%) | 1289 (80%) | 67 (76%) |
| IDU | 13 (7%) | 26 (5%) | 24 (6%) | 114 (7%) | 8 (9%) |
| Heterosexual or other | 34 (17%) | 207 (37%) | 57 (14%) | 199 (12%) | 13 (15%) |
| CD4 count, ^a^ cells/µL | 542 (405, 726) | 624 (450, 853) | 662 (469, 850) | 657 (487, 846) | 695 (531, 911) |
| HIV RNA <200 copies/mL ^a^ | 187 (94%) | 495 (89%) | 390 (93%) | 1526 (95%) | 80 (91%) |
| Insurance type |  |  |  |  |  |
| Private | 166 (84%) | 377 (68%) | 333 (79%) | 1027 (64%) | 65 (74%) |
| Medicare | 16 (8%) | 126 (23%) | 60 (14%) | 513 (32%) | 17 (19%) |
| Medicaid | 13 (7%) | 50 (9%) | 24 (6%) | 50 (3%) | 5 (6%) |
| Other | 3 (2%) | 5 (1%) | 2 (0%) | 12 (1%) | 1 (1%) |
| NDI quartile ^b^ |  |  |  |  |  |
| 1 (least deprived) | 51 (26%) | 49 (9%) | 88 (21%) | 505 (32%) | 24 (27%) |
| 2 | 52 (26%) | 116 (21%) | 108 (26%) | 428 (27%) | 22 (25%) |
| 3 | 48 (24%) | 145 (26%) | 110 (26%) | 381 (24%) | 20 (23%) |
| 4 (most deprived) | 46 (23%) | 246 (44%) | 113 (27%) | 285 (18%) | 22 (25%) |

Numbers are N (%) or median (IQR). Abbreviations: IDU, injection drug use; IQR, interquartile range; MSM, men who have sex with men; NDI, neighborhood deprivation index; TAPS, Tobacco, Alcohol, Prescription medication, and other Substance Use Tool.

^a^ Closest measurement within six months before or after screening date.

^b^ Calculated according to Messer et al. (2006) and divided in quartiles based on the distribution of the entire patient sample.
