# Supplementary material for: Racial, ethnic, and age disparities in the association of mental health symptoms and polysubstance use among persons in HIV care
Source: PLoS One. 2023 Nov 28;18(11):e0294483. doi: 10.1371/journal.pone.0294483 (PMC10684077; doi:10.1371/journal.pone.0294483)
Supplement: S3 Table — (DOCX) [file pone.0294483.s003.docx]

# S3 Table. Unadjusted and adjusted prevalence ratios (95% confidence intervals) for the association with polysubstance use, stratified by race/ethnicity, among men with HIV.

| Characteristic | Black (N=442) | | Hispanic (N=391) | | White (N=1602) | |
| --- | --- | --- | --- | --- | --- | --- |
|  | Unadjusted ^a^ | Adjusted ^b^ | Unadjusted ^a^ | Adjusted ^b^ | Unadjusted ^a^ | Adjusted ^b^ |
| Depression, anxiety, or both vs. none | 1.73 (1.34, 2.24) | 1.47 (1.11, 1.96) | 1.17 (0.84, 1.62) | 1.07 (0.74, 1.54) | 1.15 (0.91, 1.45) | 1.10 (0.85, 1.41) |
| Age, years |  |  |  |  |  |  |
| <30 | 2.12 (1.52, 2.97) | 2.29 (1.51, 3.47) | 2.40 (1.63, 3.55) | 2.30 (1.42, 3.70) | 2.19 (1.36, 3.52) | 2.41 (1.48, 3.92) |
| 30–39 | 1.53 (1.10, 2.14) | 1.56 (1.09, 2.23) | 2.14 (1.49, 3.07) | 2.01 (1.35, 3.00) | 1.91 (1.46, 2.50) | 2.02 (1.46, 2.78) |
| 40–49 | 0.97 (0.63, 1.49) | 1.00 (0.64, 1.57) | 1.17 (0.77, 1.76) | 1.14 (0.72, 1.79) | 1.42 (1.10, 1.83) | 1.48 (1.11, 1.99) |
| 50–59 | 1 (ref.) | 1 (ref.) | 1 (ref.) | 1 (ref.) | 1 (ref.) | 1 (ref.) |
| ≥60 | 0.66 (0.43, 1.01) | 0.70 (0.41, 1.19) | 0.56 (0.29, 1.07) | 0.79 (0.37, 1.69) | 0.78 (0.61, 0.98) | 0.70 (0.51, 0.96) |
| HIV risk group |  |  |  |  |  |  |
| MSM | 1 (ref.) | 1 (ref.) | 1 (ref.) | 1 (ref.) | 1 (ref.) | 1 (ref.) |
| IDU | 1.32 (0.80, 2.20) | 1.69 (1.03, 2.79) | 1.82 (1.27, 2.60) | 1.59 (1.08, 2.35) | 1.17 (0.84, 1.64) | 1.25 (0.88, 1.76) |
| Hetero./other | 0.98 (0.71, 1.34) | 1.24 (0.91, 1.71) | 0.96 (0.57, 1.64) | 1.19 (0.65, 2.18) | 1.24 (0.93, 1.65) | 1.23 (0.89, 1.69) |
| CD4 count, cells/mm^3^ |  |  |  |  |  |  |
| <200 | 0.88 (0.46, 1.66) | 0.98 (0.54, 1.78) | 0.48 (0.13, 1.71) | 0.55 (0.16, 1.88) | 0.82 (0.45, 1.50) | 0.77 (0.41, 1.45) |
| 200–350 | 1.21 (0.81, 1.82) | 1.16 (0.75, 1.79) | 1.18 (0.74, 1.88) | 1.37 (0.87, 2.15) | 0.90 (0.59, 1.36) | 1.00 (0.66, 1.52) |
| 351–500 | 0.90 (0.62, 1.32) | 1.03 (0.72, 1.46) | 0.89 (0.57, 1.41) | 0.98 (0.64, 1.51) | 0.88 (0.65, 1.18) | 0.92 (0.68, 1.24) |
| >500 | 1 (ref.) | 1 (ref.) | 1 (ref.) | 1 (ref.) | 1 (ref.) | 1 (ref.) |
| HIV RNA ≥200 vs. <200 copies/mL | 1.66 (1.20, 2.29) | 1.18 (0.82, 1.68) | 0.89 (0.43, 1.87) | 0.74 (0.29, 1.86) | 1.03 (0.53, 1.98) | 0.98 (0.51, 1.89) |
| Insurance type |  |  |  |  |  |  |
| Private | 1 (ref.) | 1 (ref.) | 1 (ref.) | 1 (ref.) | 1 (ref.) | 1 (ref.) |
| Medicare | 0.66 (0.45, 0.97) | 0.71 (0.40, 1.28) | 0.45 (0.24, 0.83) | 0.62 (0.27, 1.42) | 0.92 (0.75, 1.13) | 1.45 (1.07, 1.95) |
| Medicaid | 1.11 (0.70, 1.76) | 0.74 (0.46, 1.18) | 1.35 (0.88, 2.07) | 1.20 (0.72, 2.00) | 1.62 (1.10, 2.37) | 1.15 (0.70, 1.87) |
| Other | 2.77 (2.39, 3.21) | 2.73 (1.63, 4.58) | 1.35 (0.34, 5.45) | 1.71 (1.09, 2.68) | 0.36 (0.05, 2.36) | 0.64 (0.11, 3.59) |
| NDI quartile |  |  |  |  |  |  |
| 1 (least deprived) | 1 (ref.) | 1 (ref.) | 1 (ref.) | 1 (ref.) | 1 (ref.) | 1 (ref.) |
| 2 | 1.16 (0.64, 2.12) | 1.11 (0.63, 1.97) | 0.97 (0.66, 1.41) | 0.97 (0.61, 1.53) | 1.00 (0.79, 1.28) | 0.79 (0.60, 1.03) |
| 3 | 1.49 (0.85, 2.61) | 1.12 (0.66, 1.87) | 0.89 (0.60, 1.31) | 0.90 (0.59, 1.39) | 0.90 (0.70, 1.17) | 0.69 (0.52, 0.93) |
| 4 (most deprived) | 1.51 (0.87, 2.60) | 1.08 (0.65, 1.80) | 0.92 (0.63, 1.35) | 0.79 (0.51, 1.23) | 1.12 (0.86, 1.45) | 0.88 (0.66, 1.17) |

Abbreviations: Hetero., heterosexual; IDU, injection drug use; MSM, men who have sex with men; NDI, neighborhood deprivation index.

^a^ Estimates and 95% confidence intervals from separate Poisson regression models with generalized estimating equations. Each model includes only one characteristic.

^b^ Estimates and 95% confidence intervals from a single Poisson regression model with generalized estimating equation including all variables in the table.
