# Supplementary material for: Racial, ethnic, and age disparities in the association of mental health symptoms and polysubstance use among persons in HIV care
Source: PLoS One. 2023 Nov 28;18(11):e0294483. doi: 10.1371/journal.pone.0294483 (PMC10684077; doi:10.1371/journal.pone.0294483)
Supplement: S4 Table — (DOCX) [file pone.0294483.s004.docx]

# S4 Table. Prevalence ratios comparing the probability of polysubstance use between PWH with positive screens for depression, anxiety, or both and PWH with no positive mental health screen, unadjusted and adjusted for a single covariate at a time, with the percentage change from unadjusted estimate.

| Covariate | Black (N=442) | | Hispanic (N=391) | | White (N=1602) | |
| --- | --- | --- | --- | --- | --- | --- |
|  | PR ^a^ | Change in estimate ^b^ | PR ^a^ | Change in estimate ^b^ | PR ^a^ | Change in estimate ^b^ |
| None (unadjusted) | 1.73 | N/A | 1.17 | N/A | 1.15 | N/A |
| Age | 1.53 | 11.4% | 1.06 | 9.10% | 1.06 | 8.0% |
| HIV risk group | 1.72 | 0.8% | 1.15 | 1.50% | 1.15 | 0.3% |
| CD4 count | 1.69 | 2.6% | 1.21 | 3.60% | 1.22 | 6.2% |
| HIV RNA | 1.68 | 3.2% | 1.18 | 1.50% | 1.15 | 0.3% |
| Insurance type | 1.69 | 2.2% | 1.16 | 0.10% | 1.14 | 0.6% |
| NDI quartile | 1.78 | 2.9% | 1.16 | 0.70% | 1.14 | 1.0% |

Abbreviations: N/A, not applicable; NDI, neighborhood deprivation index; PR, prevalence ratio.

^a^ Estimates and 95% confidence intervals from separate Poisson regression models with generalized estimating equations. Each model includes only the covariate listed in the left column.

^b^ Calculated as |(adjusted PR - unadjusted PR) / unadjusted PR| and expressed as a percentage.
