# Supplementary material for: Racial, ethnic, and age disparities in the association of mental health symptoms and polysubstance use among persons in HIV care
Source: PLoS One. 2023 Nov 28;18(11):e0294483. doi: 10.1371/journal.pone.0294483 (PMC10684077; doi:10.1371/journal.pone.0294483)
Supplement: S5 Table — (DOCX) [file pone.0294483.s005.docx]

# S5 Table. Polysubstance use prevalence and prevalence ratios comparing persons with HIV by mental health screening results, in a sensitivity analysis using a TAPS score ≥2.

| Positive mental health screen ^a^ | N | Polysubstance Use Prevalence ^b^ (95% CI) | Unadjusted PR (95% CI) ^c^ | Adjusted PR (95% CI) ^d^ |
| --- | --- | --- | --- | --- |
| Depression, anxiety, or both | 515 | 15.7% (12.9%, 19.2%) | 2.62 (2.03, 3.39) | 2.05 (1.54, 2.72) |
| Neither | 2350 | 6.0% (5.1%, 7.0%) | 1 (ref.) | 1 (ref.) |

Abbreviations: CI, confidence interval; PR, prevalence ratio.

^a^ Depression was defined as a PHQ-9 score ≥10. Anxiety was defined as a GAD-2 score ≥3.

^b^ Polysubstance use was defined as having a score ≥2 for two or more substances on the Tobacco, Alcohol, Prescription medication and other Substance use (TAPS) Tool.

^c^ Estimates and 95% CIs were obtained from a Poisson regression model with robust variance.

^d^ Adjusted for sex, race, ethnicity, age, HIV risk group, HIV viral load, CD4 count, Neighborhood Deprivation Index (NDI), and insurance.
