# Supplementary figures and images for: Racial, ethnic, and age disparities in the association of mental health symptoms and polysubstance use among persons in HIV care
Source: PLoS One. 2023 Nov 28;18(11):e0294483. doi: 10.1371/journal.pone.0294483 (PMC10684077; doi:10.1371/journal.pone.0294483)

# S1 Figure. Flowchart of patient inclusion.


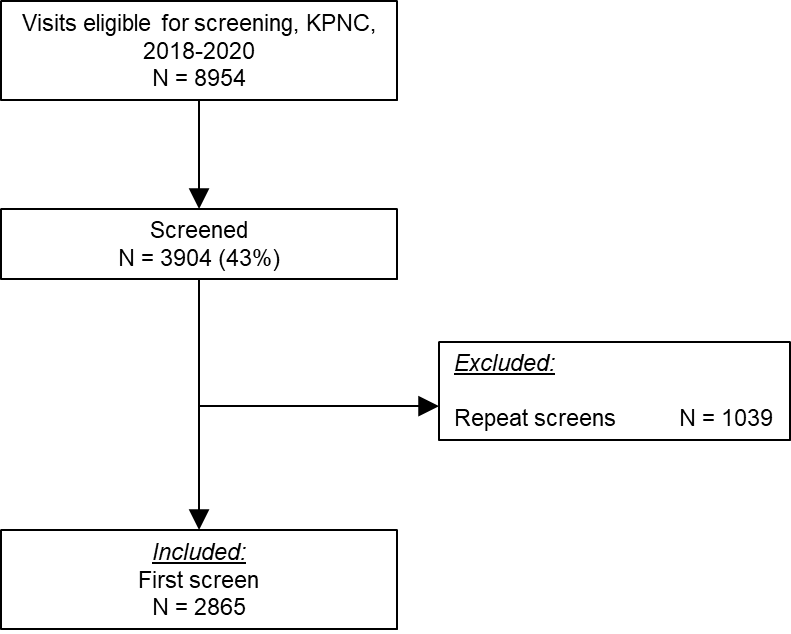

Supplement: S1 Fig — (DOCX) [file pone.0294483.s006.docx]
