# Supplementary material for: Racial, ethnic, and age disparities in the association of mental health symptoms and polysubstance use among persons in HIV care
Source: PLoS One. 2023 Nov 28;18(11):e0294483. doi: 10.1371/journal.pone.0294483 (PMC10684077; doi:10.1371/journal.pone.0294483)

# S2 Figure. Prevalence of polysubstance use by clinical and demographic characteristics in a sensitivity analysis using a TAPS score ≥2. Abbreviations: API, Asian or Pacific Islander; Hetero., heterosexual; IDU, injection drug use; MSM, men who have sex with men; NDI, neighborhood deprivation index. * denotes *P* <0.05.


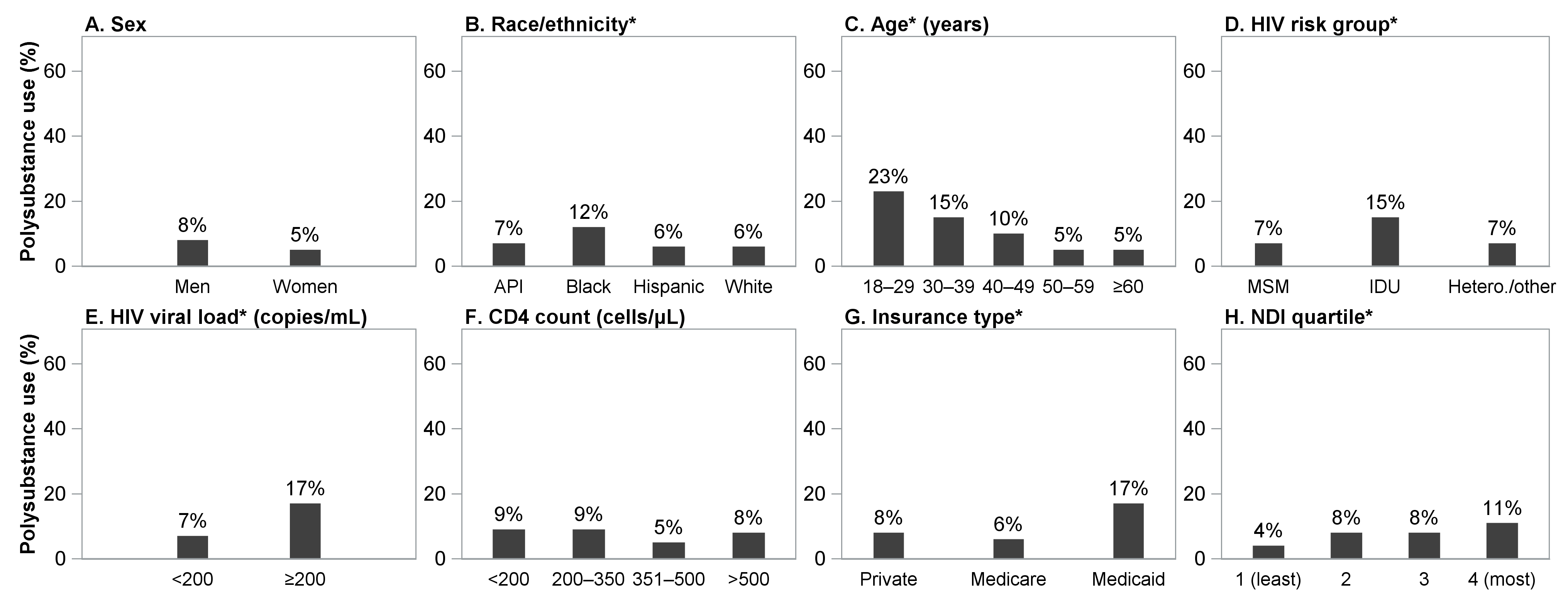

Supplement: S2 Fig — Abbreviations: API, Asian or Pacific Islander; Hetero., heterosexual; IDU, injection drug use; MSM, men who have sex with men; NDI, neighborhood deprivation index. * denotes P <0.05. (DOCX) [file pone.0294483.s007.docx]
