# Supplementary material for: Racial, ethnic, and age disparities in the association of mental health symptoms and polysubstance use among persons in HIV care
Source: PLoS One. 2023 Nov 28;18(11):e0294483. doi: 10.1371/journal.pone.0294483 (PMC10684077; doi:10.1371/journal.pone.0294483)

# S3 Figure. Prevalence of polysubstance among patients screening positive for either depression or anxiety versus neither, stratified by (A) race and ethnicity and (B) age, in a sensitivity analysis using a TAPS score ≥2.


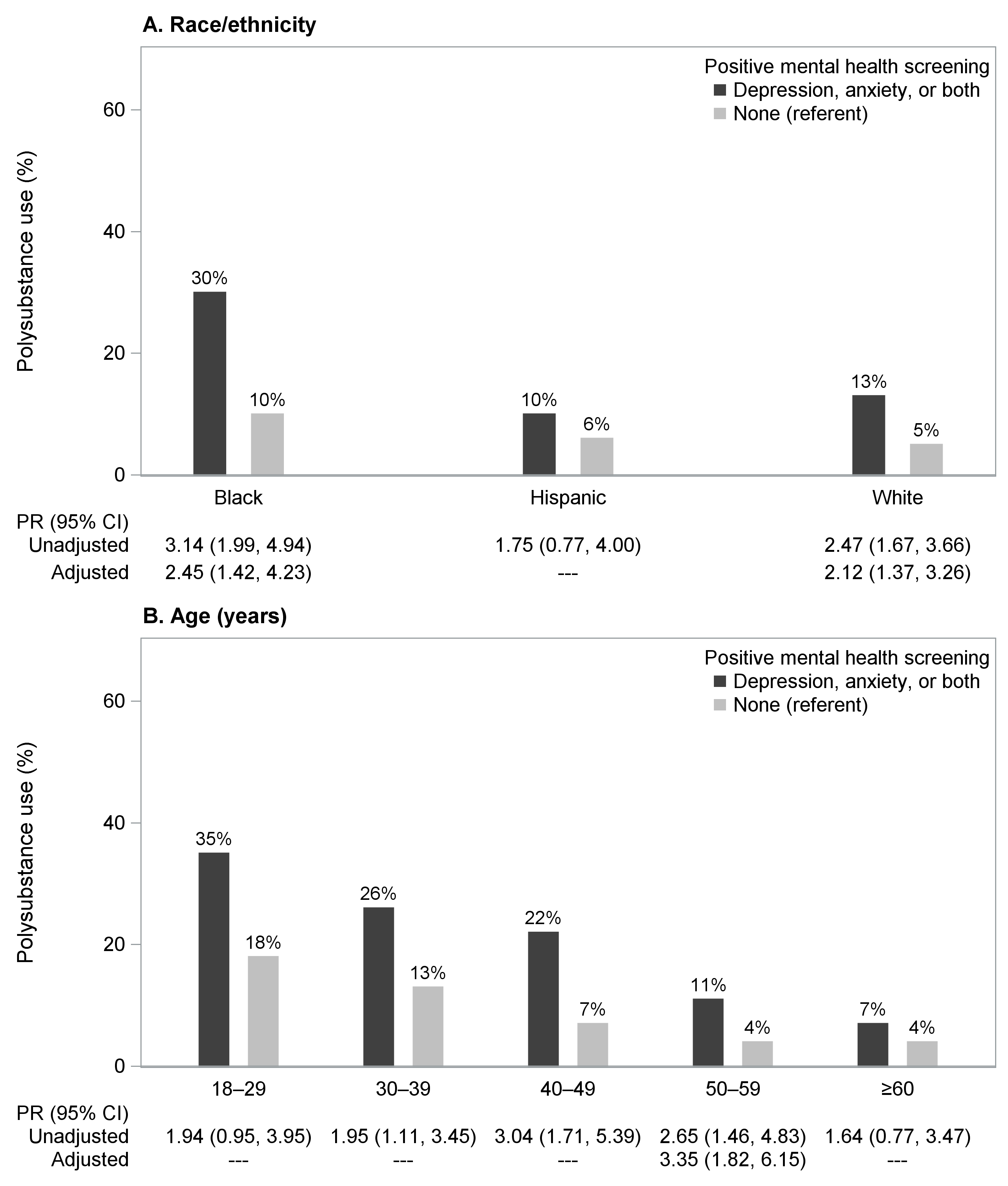

Supplement: S3 Fig — Prevalence of polysubstance among patients screening positive for either depression or anxiety versus neither, stratified by (A) race and ethnicity and (B) age, in a sensitivity analysis using a TAPS score ≥2. (DOCX) [file pone.0294483.s008.docx]
